# Supplementary material for: Mechanical coupling in the nitrogenase complex
Source: PLoS Comput Biol. 2021 Mar 4;17(3):e1008719. doi: 10.1371/journal.pcbi.1008719 (PMC7963043; doi:10.1371/journal.pcbi.1008719)
Supplement: S1 Table — (DOCX) [file pcbi.1008719.s001.docx]

**S1 Table. Crystal structures of the ADP- and ATP-bound nitrogenase complexes.**

|  |  | ATP-bound  (2AFK) | ADP-bound  (2AFI [I]) |
| --- | --- | --- | --- |
| Number  of residues solved in the crystal structures | α_1_ of MoFe protein | 477 | 476 |
|  | β_1_ of MoFe protein | 522 | 522 |
|  | α_2_ of MoFe protein | 476 | 476 |
|  | β_2_ of MoFe protein | 522 | 522 |
|  | γ_1_ of Fe protein 1 | 270 | 271 |
|  | γ_2_ of Fe protein 1 | 263 | 275 |
|  | γ_1_ of Fe protein 2 | 272 | 263 |
|  | γ_2_ of Fe protein 2 | 262 | 269 |
| RMSD of Cα (Å)^a^ | α_1_β_1_ of MoFe protein (998*) | 0.3 | |
|  | α_2_β_2_ of MoFe protein (998*) | 0.3 | |
|  | Fe protein 1 (533*) | 2.7 | |
|  | Fe protein 2 (532*) | 2.3 | |
|  | The whole complex | 6.6 | |

^a^ RMSD calculations compared residues present in both crystal structures; the number of residues that appear in both structures is indicated (*). Structures are aligned on the corresponding components.
