# Supplementary figures and images for: Mechanical coupling in the nitrogenase complex
Source: PLoS Comput Biol. 2021 Mar 4;17(3):e1008719. doi: 10.1371/journal.pcbi.1008719 (PMC7963043; doi:10.1371/journal.pcbi.1008719)

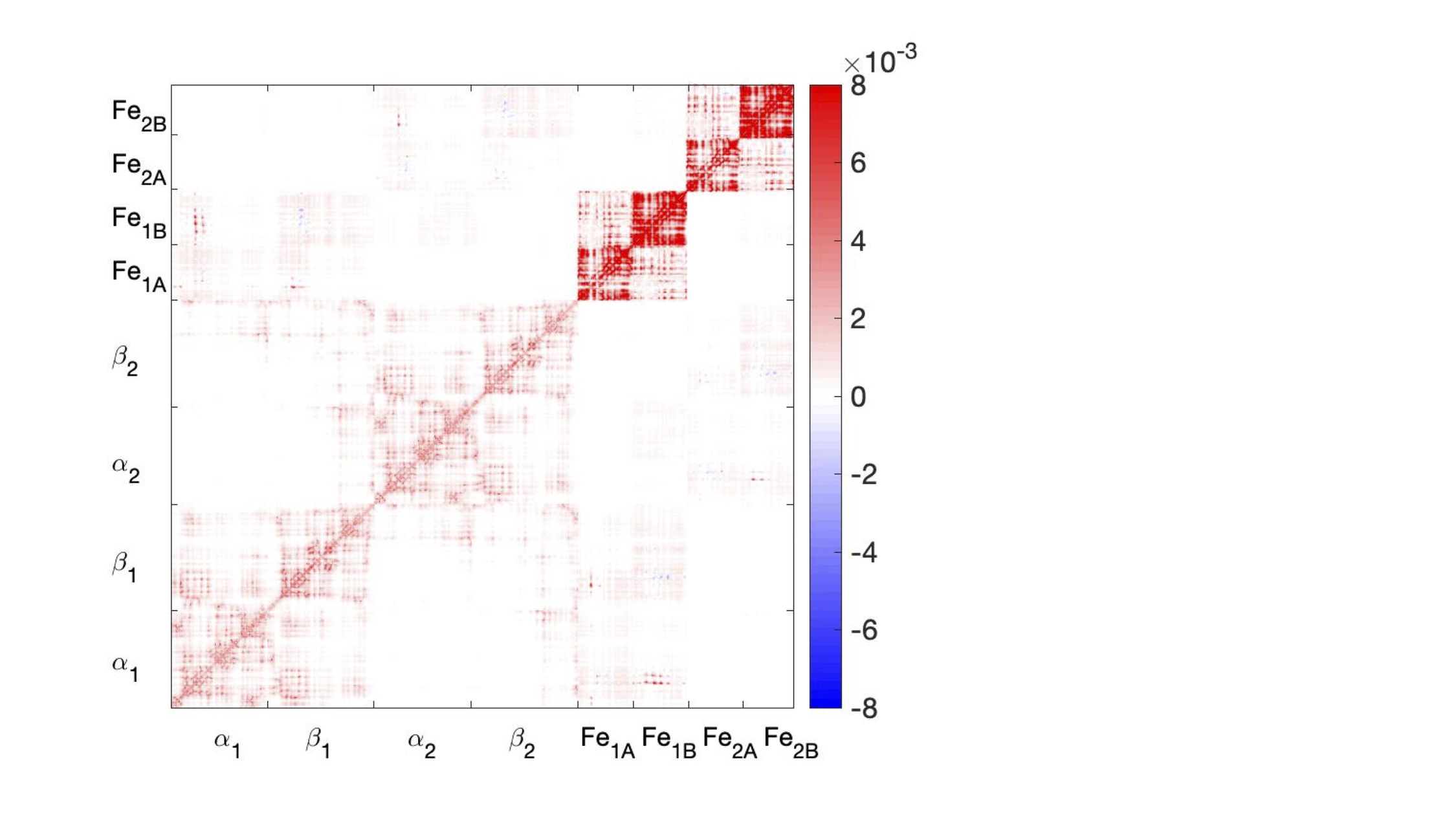

Supplement: S1 Fig — (TIF) [file pcbi.1008719.s002.tif]

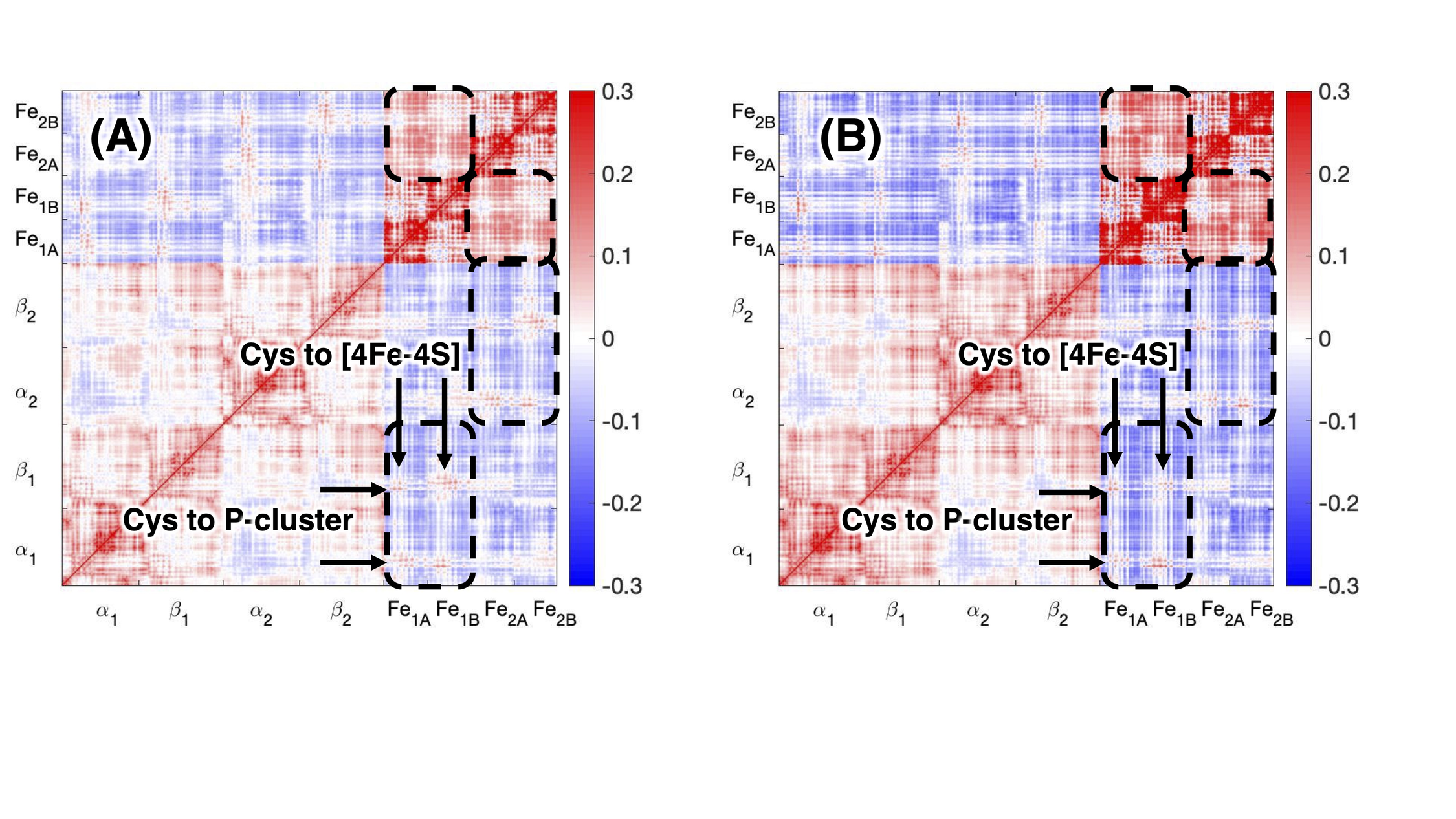

Supplement: S2 Fig — Normalized correlation matrix C of the residues displacements for (A) the ATP-bound nitrogenase complex and (B) the ADP-bound nitrogenase complex. The correlation between Fe1 and α1β1, Fe2 and α2β2, Fe1 and Fe2 are highlighted in dashed blocks. The locations of the cysteine ligands to the [4Fe-4S] cluster in Fe1 and P-cluster in α1β1 are indicated by arrows in correlation between Fe1 and α1β1. (TIF) [file pcbi.1008719.s003.tif]

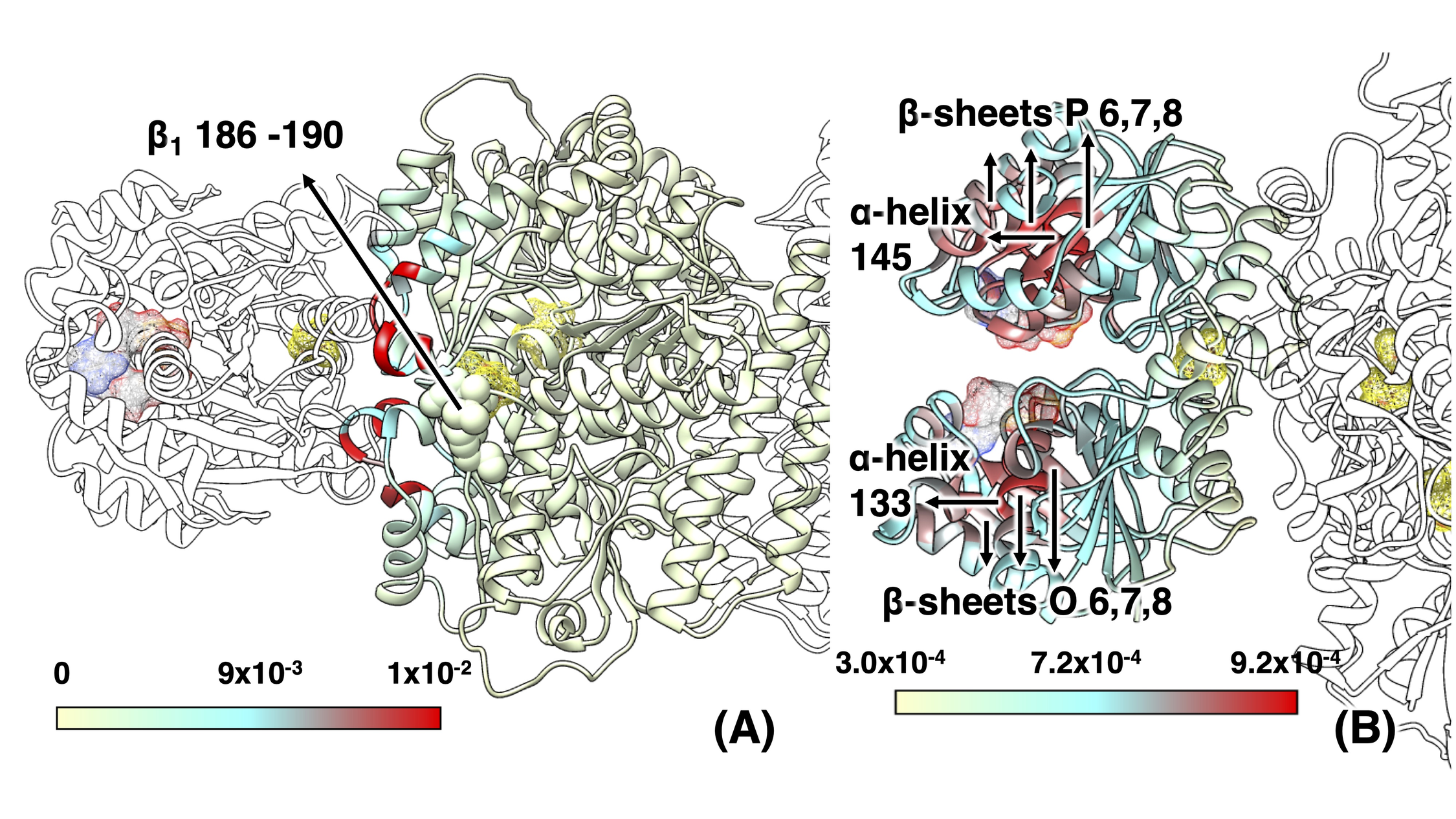

Supplement: S3 Fig — (A) residues in α1β1 to any residue in Fe1 and (B) residues in Fe2 to any residue in Fe1 in ADP-bound complex. The ATP analogues, [4Fe-4S] clusters, P-clusters, and FeMo-co are shown in mesh surface. Residue 186–190 in β1 are shown in spheres in (A); one α-helix and three β-sheets in each monomer of Fe2 appear red in (B) (following the numbering in the 2AFI [I] PDB file). (TIF) [file pcbi.1008719.s004.tif]

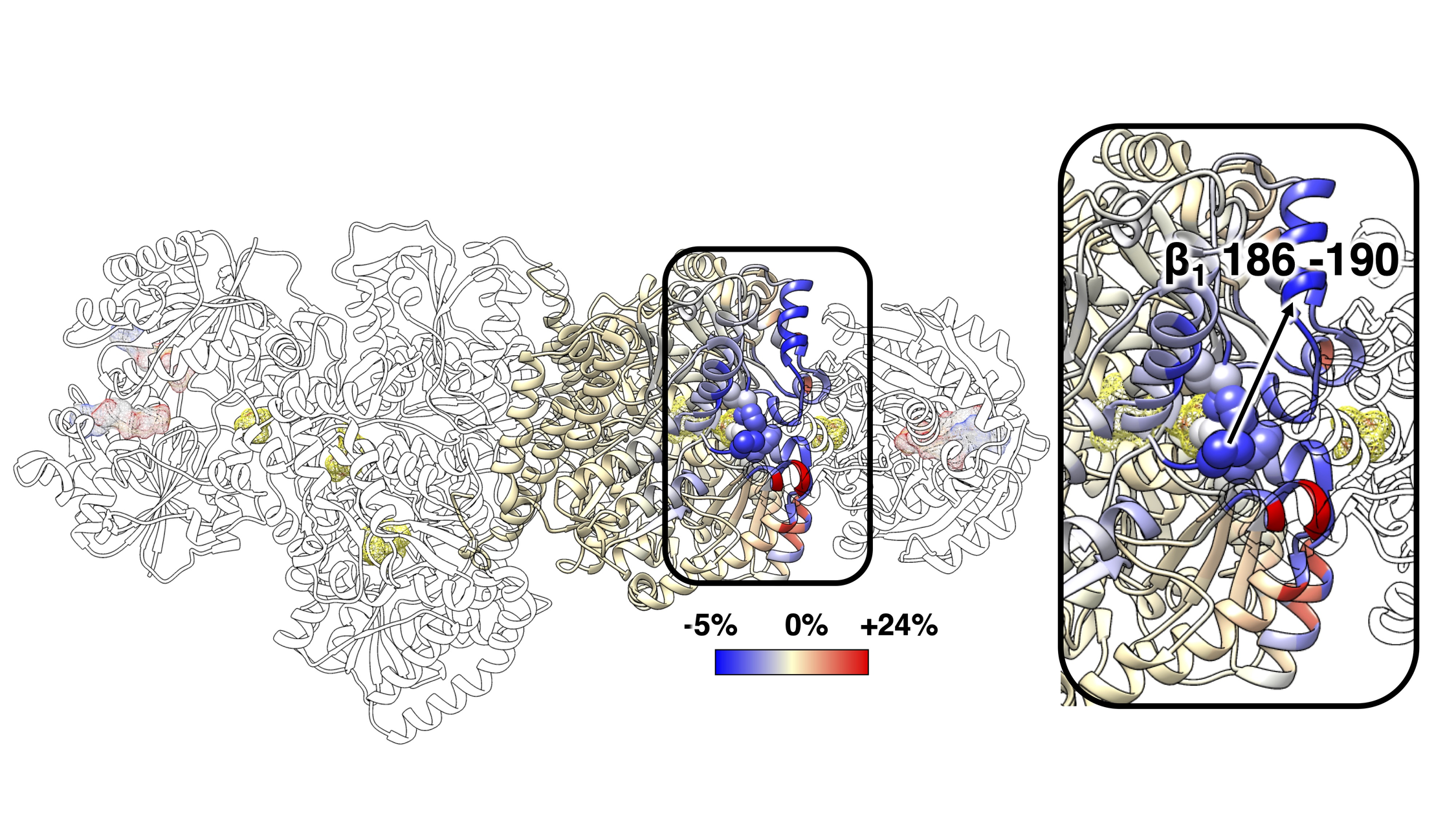

Supplement: S4 Fig — The change is shown as largest deviation of M in the ADP-bound complex relative to the ATP-bound complex, either positive or negative. The Fe1, Fe2, α2β2 proteins are shown in white. The ATP analogues, [4Fe-4S] clusters, P-clusters, and FeMo-co are shown as mesh surfaces. Residues 186–190 in β1 are highlighted in spheres. Residues at Fe protein/MoFe protein interface with large deviation upon ATP hydrolysis appear in dark red or dark blue. (TIF) [file pcbi.1008719.s005.tif]

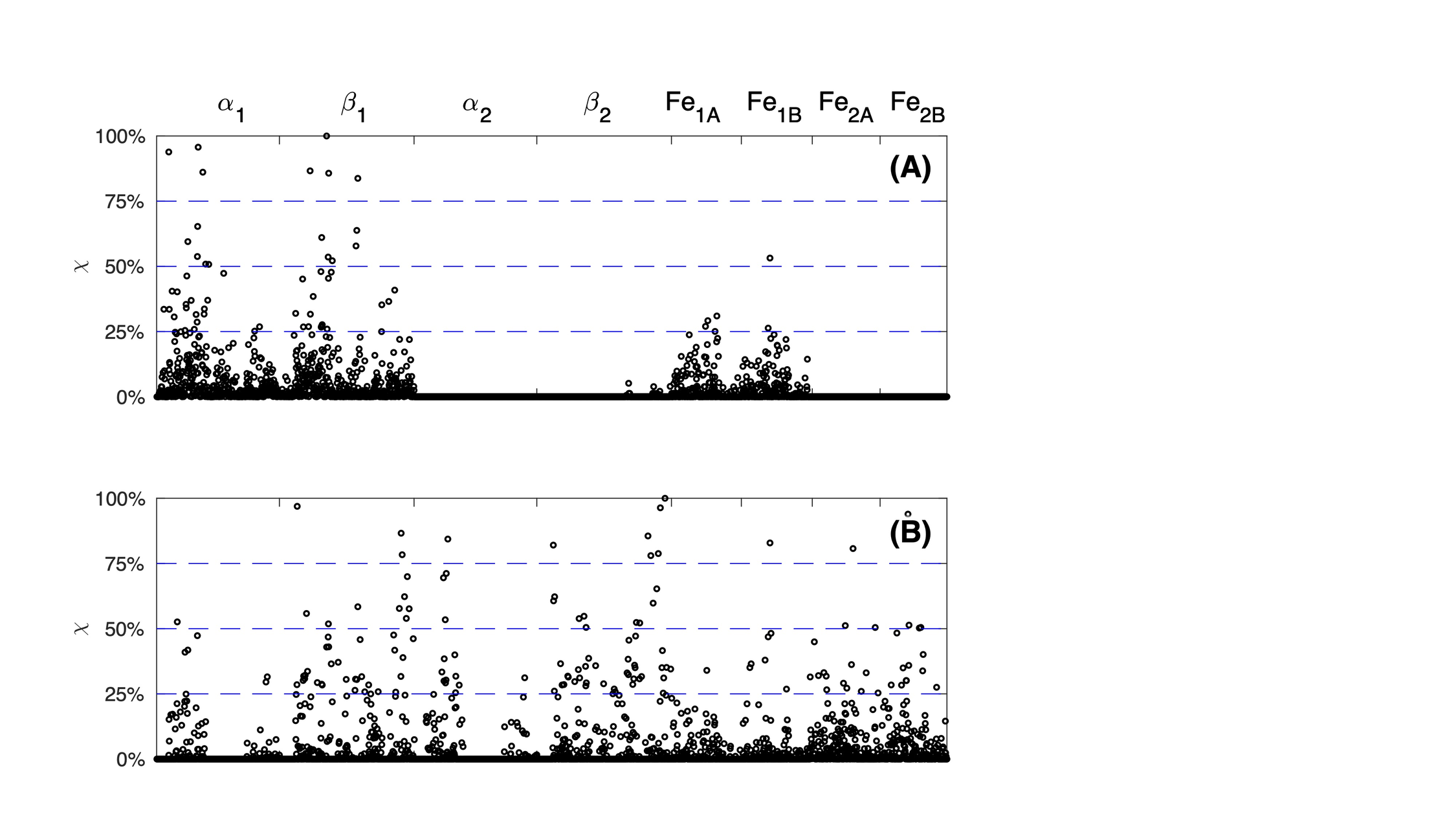

Supplement: S5 Fig — (A) X = Fe1 and Y = α1β1; (B) X = Fe1 and Y = Fe2. Each circle represents a residue. χ is shown as the percentage relative to the corresponding maximum χX-Y. (TIF) [file pcbi.1008719.s006.tif]

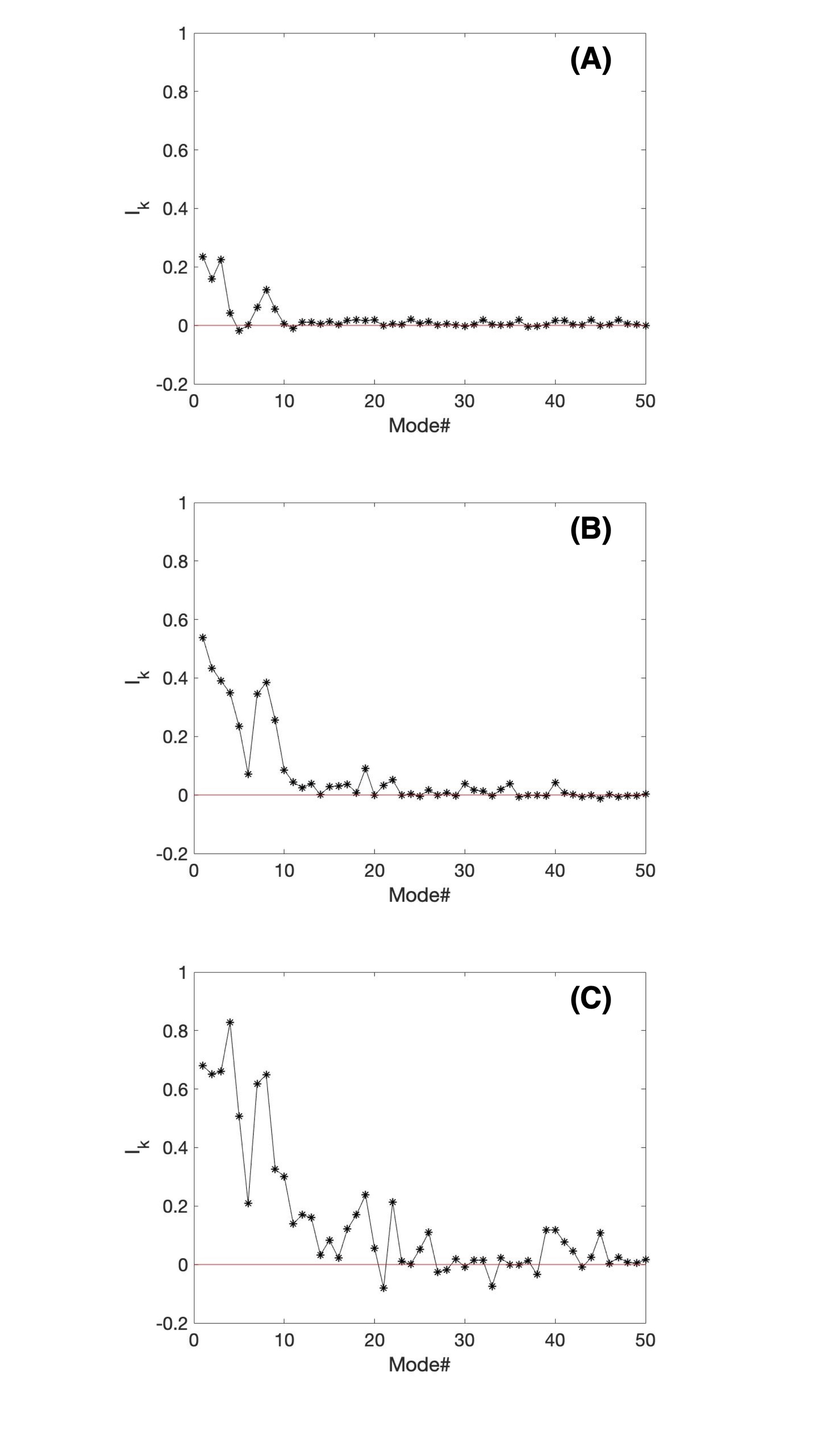

Supplement: S6 Fig — (A) X = Fe1 and Y = α1β1; (B) X = Fe1 and Y = Fe2, (C) X = P-loops in Fe1 and Y = P-loops in Fe2. (TIF) [file pcbi.1008719.s007.tif]

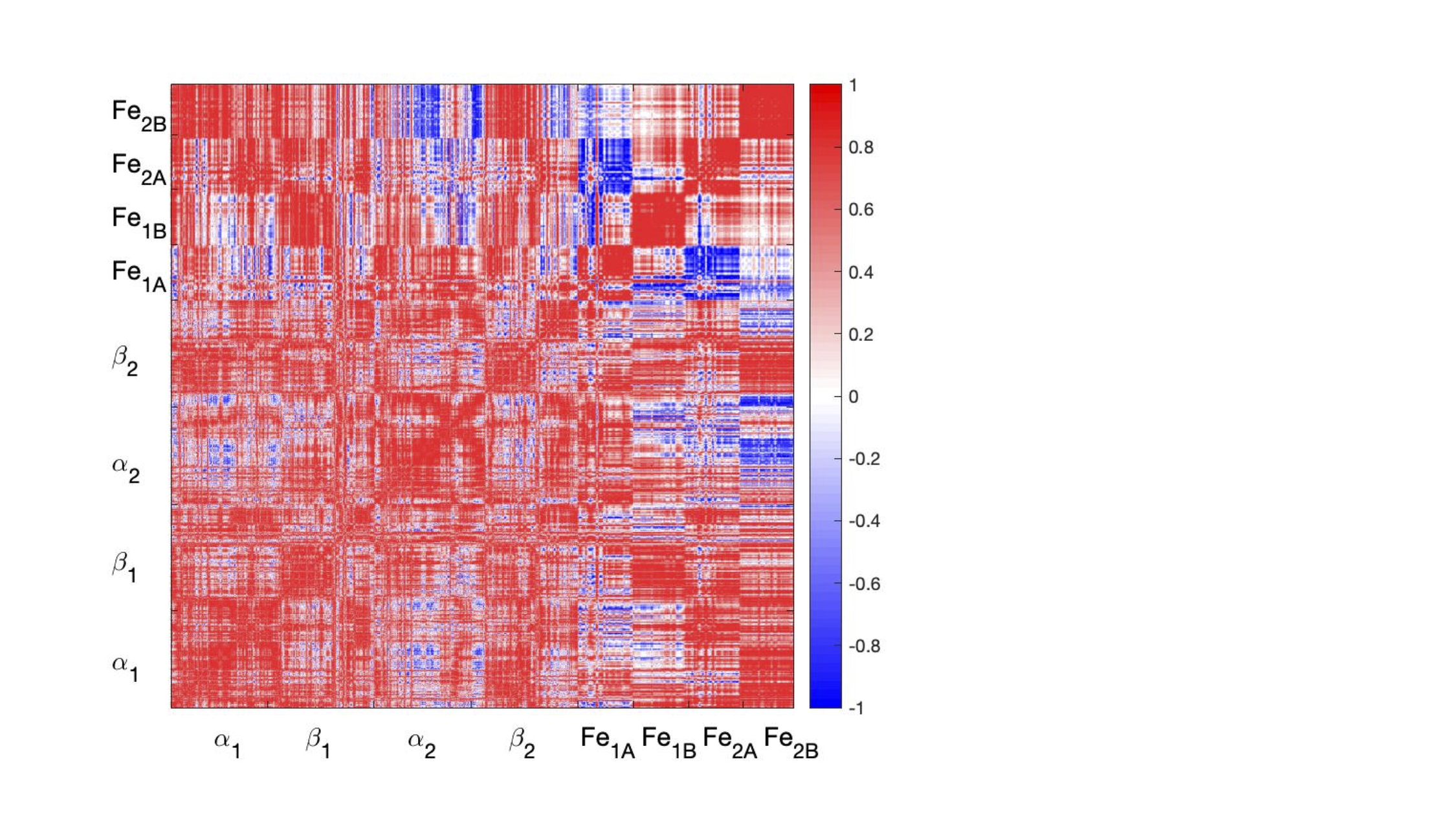

Supplement: S7 Fig — (TIF) [file pcbi.1008719.s008.tif]

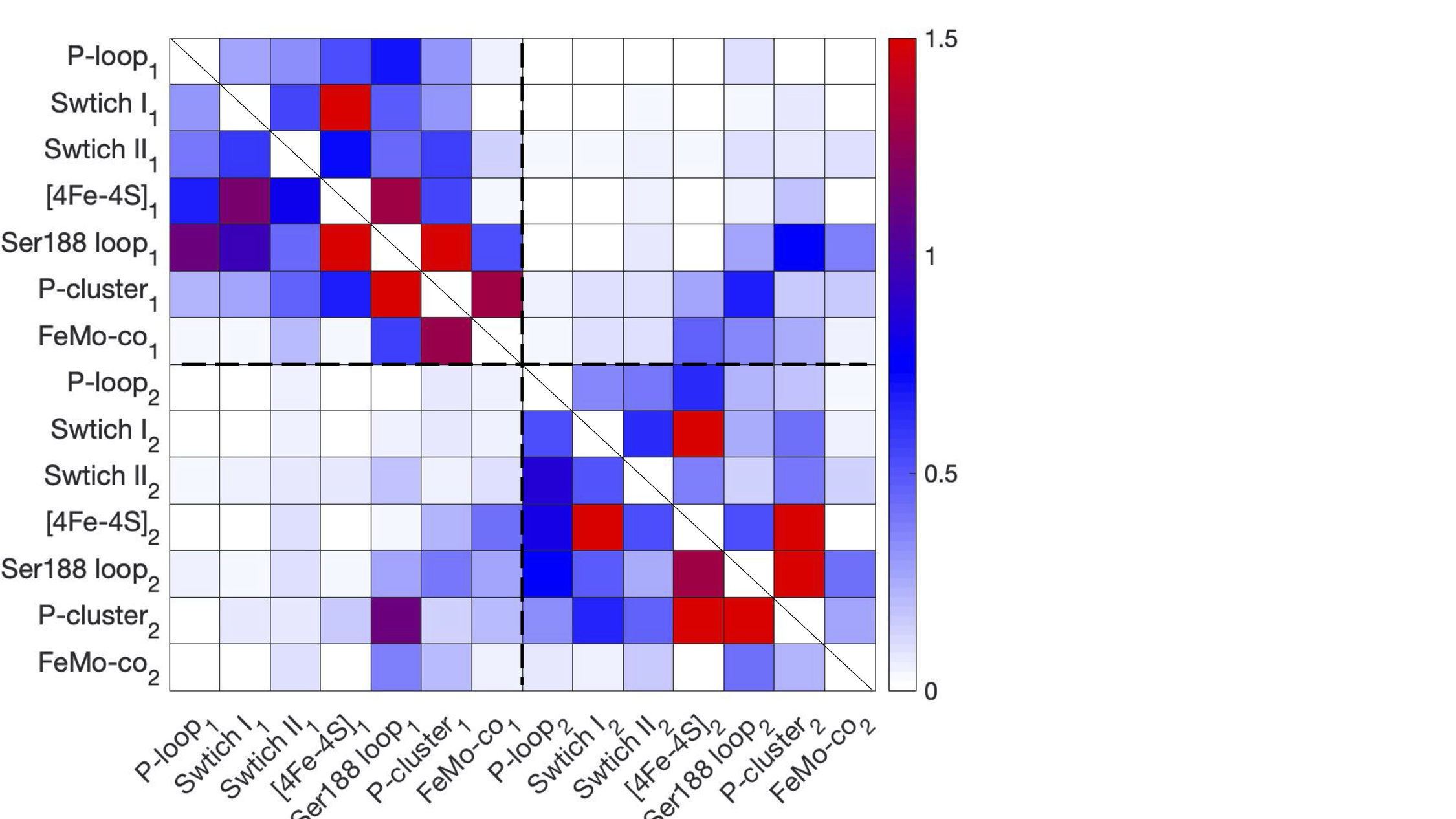

Supplement: S8 Fig — Overlaps of coupling pathways between structural elements X and Y, i.e., OX-Y, between the motions of the ATP-bound complex as obtained from ANM and the ATP- to ADP-bound complex atomic displacement as inferred from the corresponding crystal structures are shown. The subscript of the X or Y elements indicates which half of the complex the element belongs to. (TIF) [file pcbi.1008719.s009.tif]

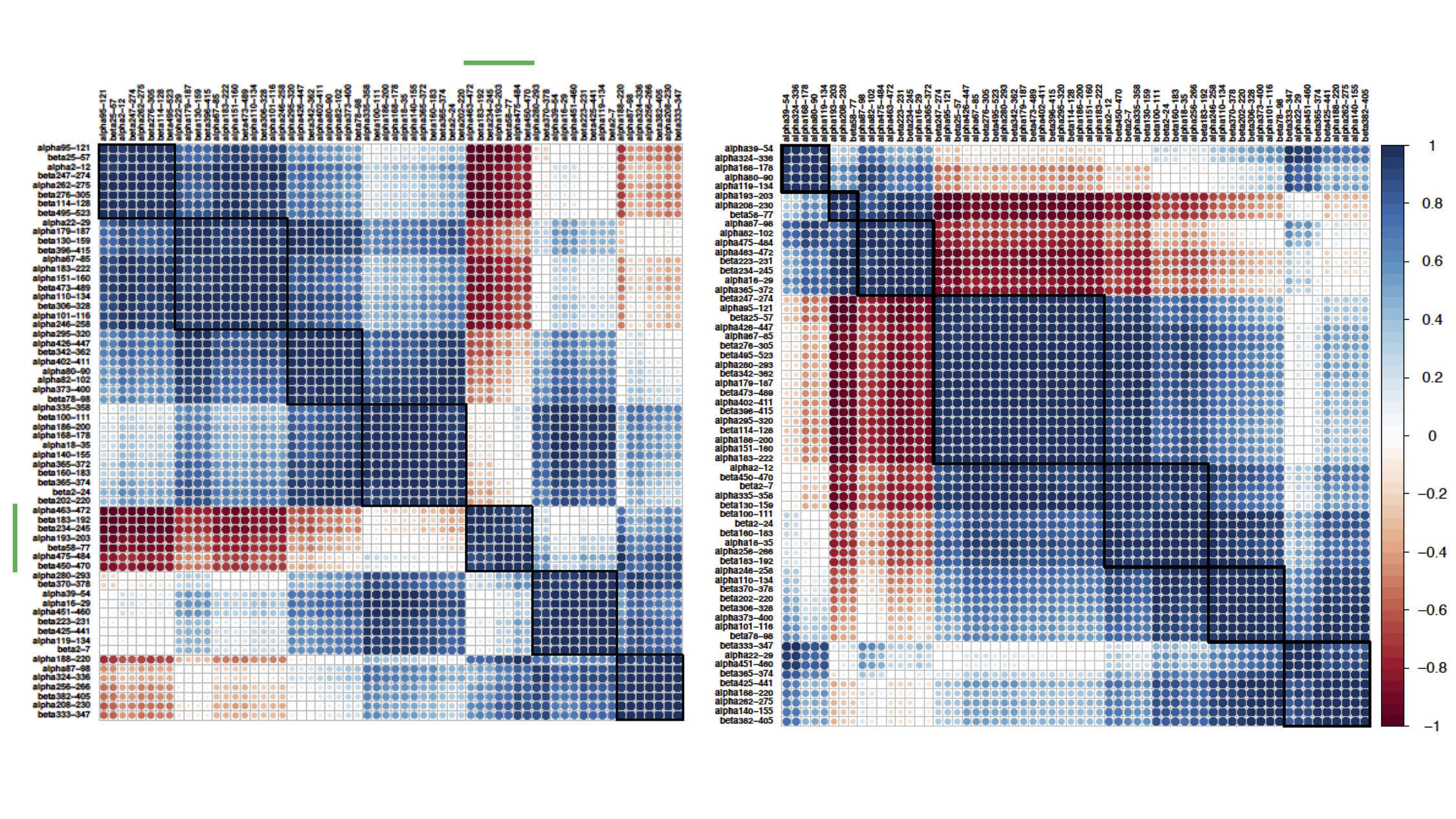

Supplement: S9 Fig — Red and blue circles correspond to negative and positive correlations, respectively. White indicates no correlation. Diameter of the circle represents significance; larger is more significant correlation. Peptides are group based on hierarchical clustering. Green bar indicates peptides which are negatively correlated in free MoFe protein. (TIF) [file pcbi.1008719.s010.tif]
