# Supplementary material for: Mechanical coupling in the nitrogenase complex
Source: PLoS Comput Biol. 2021 Mar 4;17(3):e1008719. doi: 10.1371/journal.pcbi.1008719 (PMC7963043; doi:10.1371/journal.pcbi.1008719)
Supplement: Attachment — Submitted filename: Response_20201103.pdf [file pcbi.1008719.s011.pdf]

Response to the Reviewers

Comments to the Authors:
Please note here if the review is uploaded as an attachment.

Reviewer #1: Allosteric regulation in the nitrogenase complex is studied in this work by means of graph theory analysis. The motivation for this work is the lack of complete understanding of the mechanism of allosteric communication at different stages of the protein functional cycle. There are partial data on local conformational changes and indirect evidences on the effect of mutations on functional sites of the complex. All the above prompted authors to explore the mechanical coupling between the protein sites, using graph theoretical approach developed in the group.

The authors demonstrate "mechanical coupling" between functionally involved regions, as well as analyze significance of individual normal modes contributions to the coupling. They also support their calculations with the data from HDX experiments. The major caveat of the approach is that it is based on the analysis of correlated motions, while correlation does not necessarily causality. Further, they claim that regions with "highly correlated or anti-correlated motions are mechanically coupled through efficient energy transfer path". This conclusion seem to be unjustified as in the condensed matter of the protein there are no selected channels of the energy transformation, and all work produced in the structure is a result of multibody interactions between amino acid residues. If authors should resort to network consideration of protein, they should not be mixing it with the claims on the analysis of the protein energetics. If they decide to consider the causality and energetics, they may use, for example, AlloSIgMA web-server (PMID: 32392302, PMID: 29106449), allowing to calculate the allosteric free energy as a result of the perturbationand to estimate effects of mutations (PMID: 26939022, PMID: 30827842).

We certainly agree with the Reviewer that correlation does not necessarily imply causality. We thank the reviewer for suggesting the AlloSIgMA web server for causality and energetics and the papers associated with it. We will certainly consider them in forthcoming studies of the nitrogenase complex.

Here we would like to reiterate that the method we have developed was designed to filter out pairs of residues whose covariant motion is purely coincidental and evaluate potential communication pathways between distant region of a protein complex. The major difference between our M-matrix analysis and a simple covariance analysis of motion, is that if two residues have correlated motions but do not have an efficient path for mechanical coupling between them (large geodesic distance in the covariance space), a small (poorly coupled) mechanical coupling (as described by the M matrix) will be produced. In this way, geodesic weighting filters out motions that are merely coincidental. Clearly, the existence of a pathway is a necessary but not sufficient condition for causality. This is actually an important point, which was added in the revised manuscript (page 23).

“We like to emphasize that correlation or communication here does not imply causality. In fact, strong mechanical coupling indicates an efficient coupling pathway connecting two residues or regions which show correlated or anti-correlated motions. Clearly, the existence of a pathway is a necessary but not sufficient condition for causality. A study of effects of suppression or delay of the motion of one region on the motion of the other will be necessary to consider causality.”

We also realize that the language adopted in the manuscript was not clear. In particular, as originally discussed, the concept of energy transfer could have led to misinterpretations. We used the term energy transfer to indicate instantaneous flow of kinetic (thermal) energy mediated by protein vibrations (normal modes). We have revised and consolidated the language to address this point. For example, where appropriate, “energy” is changed to “information” and “energy flow” changed to “communication”.

The HDX data is of interest, but should be better linked to results of calcualtions and further discussed.

We agree that the strong correlation between the solution phase biophysical analysis using HDX and a purely computational approach are of interest and strengthen our model. The H/D exchange measurements show that regions with correlated H/D exchange patterns correspond to regions computationally identified as important for the Fe protein/MoFe protein coupling. We also agree that we failed to thoroughly explain and connect the H/D exchange results to the computational analysis. We have added to the results section to amend this. For example, we moved the extended outline from Figure S9 legend to the results section in order to improve flow of provided information and highlight a holistic aspect of our analysis (page 19). That paragraph now reads:

“To investigate the effects of the presence of Fe protein on long-distance communication of MoFe protein, we searched for regions with correlated patterns of H/D exchange. Based on the difference in percent deuterium exchange between regions, we built a correlation matrix for free MoFe protein and MoFe protein in the presence of ATP-bound Fe protein (S9. Fig). After one hour of exchange, seven well-defined correlation groups were defined for each condition. However, the exchange patterns and peptides in the correlation groups were not the same between conditions. In MoFe protein alone, about 62% of investigated peptides show strong positive correlation (blue), while 22% have a strong negative correlation (red). In the presence of ATP-bound Fe protein, MoFe protein exhibits much greater negative correlation (36%). In MoFe alone, only peptides in the fifth cluster (green bars) have significant negative correlation. These peptides are spread across structure including the MoFe protein dimer interface, near Fe protein binding site, P-cluster and FeMo-co. Enhanced negative correlation upon binding of Fe protein is consistent with a model of negative cooperativity between “halves” of MoFe protein.”

We also added another paragraph to the results section of “Testing of the mechanical coupling model” (page 21) that links the computational model with the observation derived from H/D exchange experiments. The new paragraph reads:

"Detailed analysis of theoretical and experimental data made it immediately evident that regions identified as important for the Fe protein/MoFe protein coupling in the computational analysis correspond (or are adjacent) to regions with high H/D exchange correlation. In addition, our analysis revealed that the region surrounding the P-cluster is sensitive to the presence of ATP-bound Fe protein. This agrees with observed structural changes when nitrogenase is poised at different steps in the catalytic cycle. Of particular interest is cross-talk between β-69^{Ala} and β-188^{Ser}, two resides proximal to the P-cluster ligation site. In free MoFe protein, these residues are positively correlated. Upon binding of ATP-bound Fe protein, β-69^{Ala} and β-188^{Ser} (along with the P-cluster environment in the β subunit) become negatively correlated. At the same time, H/D exchange of Ala 69 is positively correlated with the P-cluster environment in the opposite half of MoFe protein. Equally interesting is the relationship of β-188^{Ser}/β-69^{Ala} protein dynamics with $\alpha_1\beta_1$-$\alpha_2\beta_2$ interface (residues β-479^{Leu}/β-497^{Leu}). Despite spatial separation, the P-cluster ligation site and the $\alpha_1\beta_1$-$\alpha_2\beta_2$ interface show a strong correlation. However, only β-188^{Ser} is sensitive to the presence of ATP-bound Fe protein. In summary, our H/D exchange experiments reveal correlated patterns of dynamics precisely where computationally derived mechanical coupling networks predict."

The discussion is too lengthy, sometimes too speculative when results on mechanical coupling are interpreted in terms of the energy/electron transfer.

We recognize that the discussion was indeed a bit verbose and we revised it accordingly. As discussed above, we clarified our use of the concept of energy transfer.

"Important allosteric centers" is a bad phrasing.

We changed it to "important for allosteric communication"

Figures 2 and 3 should be merged in one, or Figure 2 should be moved to supplementary. Figure 9 - to supplementary as well.

The original Figure 2 and 9 are moved to SI; now Figure S2 and S8.

Reviewer #2: The authors describe an engaging and comprehensive study of synchronized and allosteric motion of the nitrogenase proteins. Importantly, the theoretical studies are connected to isotope effect experiments, and the entire body of work is place into an appealing modern perspective. While this form of modeling is simple and fast, it can be extremely useful and I believe that the paper will prove to be of great value and interest to researchers in the field. There was an early study of Liao and Beratan that reported coarse grained normal mode analysis of the nitrogenase proteins as well (see Biophysical Journal 87 1369–1377 (2004)). It would be interesting to compare the results of the two studies and to place the early albeit less comprehensive study into the context of this modern analysis, if possible.

We thank the Reviewer for pointing out the work by Liao and Beratan. We compare their results with ours in the Results and Discussion of the revised manuscript (page 12).

"Within each Fe protein, the P-loops are coupled to the protein-protein interface and the [4Fe-4S] cluster through the switch region I and II, respectively. Liao and Beratan discussed this coupling in their computational analysis of the isolated Fe protein. Using a coarse-grained model, they identified correlated regions in the Fe protein and reported that residues in the P-loops and switch regions are relatively rigid in the slowest normal mode, which can be important for function-relevant conformational changes. As we will discuss below, we show that there is indeed an efficient communication pathway between these regions, which, in the nitrogenase complex, extends up to the interior of the MoFe protein."

Reviewer #3: Huang et al. identified communication pathways in the nitrogenase complex comprised of two Fe and two FeMo-co proteins. Nucleotide hydrolysis in the Fe- protein initiates a long-range electron transfer from the Fe-proteins to the FeMo-co. Electron transfer to the active site of FeMo occurs by intermediate electron transfer steps through the 4Fe-4S clusters and P-cluster. It is known (also mentioned by the authors) that the nucleotide hydrolysis and the consequent electron transfer is coupled with some structural rearrangements, especially at the interface of the Fe and two FeMo-co proteins. Structures of the complex before and after the hydrolysis are available. These two endpoints indicate that the structural changes may involve a rigid body movement of the Fe-proteins. Additionally, electron transfer in one subunit slows down the electron transfer in the other subunit. The mechanism of communication is unknown. The authors performed a sophisticated network analysis using just these two structures as inputs to unravel the mechanistic pathway.

The paper presents a very sophisticated analysis for an input of just two structures. However, in my experience, it is often the case that pathways connecting two endpoint structures require further optimization as local dynamics may enhance or diminish parts of the path. Can the authors comment on this possibility?

The reviewer raised a truly important point. The comment has given us the opportunity to elaborate on the limitation of the present computational scheme in addressing how the conformational dynamics influences the mechanical coupling. In the original manuscript, we simply noted (page 18) that "potential transient large-amplitude structural changes, postulated to be relevant for the electron transfer, are not accessible through the present computational framework." In the revised manuscript we have expanded that part as follows: "The present computational framework is based on well-defined equilibrium (crystallographic) structures. Therefore, it cannot address the effect of transient structural changes, which can either enhance or weaken the relevance of the coupling pathways and, consequently, change the χ value associated with a given residue or region. More importantly, potential transient large-amplitude structural changes, postulated to be relevant for the electron transfer, are clearly not accessible through a Gaussian model."

Additionally, the 2AFK structure (used by the author) has been made obsolete and replaced in the PDB by 4WZB. Can the authors compare the structures to ensure something wasn't incorrect in the older, obsolete one?

We thank the reviewer for raising this point. The major difference between the 4WZB and 2AFK structures is the nature of the FeMo-co central atom (C vs. N). The position of the Cα atoms, which is used in our normal mode analysis, is very similar in the two structures (RMSD=0.18 Å). We adopted the 2AFK pdb structure for modeling the ATP-bound complex for consistency with the structure adopted for the ADP-bound complex (2AFI pdb structure). We added a note in the Methods to clarify this point (page 6):

"While the 2AFK structure is now replaced by 4WZB, the major difference between the 4WZB and 2AFK structures is the nature of the FeMo-co central atom (C vs. N). The position of the Cα atoms, which are used in our normal mode analysis, is very similar in the two structures (RMSD=0.18 Å). We adopted the 2AFK structure for modeling the ATP-bound complex for consistency with the structure adopted for the ADP-bound complex (2AFI structure),"

The betweenness centrality used here relies on the shortest-path connecting distant domains. However, an allosteric path does not always take the shortest path through the protein systems. Sometimes, depending on specific residues, a slightly longer path may be more effective for communication, especially when electron transfer is involved. The effect of the distance can be offset by the exponential energetic dependence of the electron transfer rates. Do the authors agree with this possibility? If so, can they extend their analysis to consider pathways other than the shortest path?

We fully agree with the Reviewer and we realized, after reading his or her comment, that we were not sufficiently clear with what we meant with "shortest path". As used here, the term "shortest path" refers to the geodesic distance in covariance space and not to the geometric distance in the Cartesian space. We use the geodesic distance in the covariance space to measure the strength of mechanical coupling. That said, due to the elongated shape of the nitrogenase complex, it turns out that for the long-range communication (e.g., Fe protein/Fe protein communication), the shortest geodesic pathway, under most circumstances, is rather similar to the pathway with shortest cartesian distance between two regions. We have clarified this point in the revised manuscript (page 7).

"We indicate the shortest path in the covariance space as the most efficient pathway between *i* and *j*. Because of the elongated shape of the nitrogenase complex, the shortest path in the covariance space connecting distant parts of the complex is often similar (but not identical) to the shortest path in the cartesian space."

I'm unclear on how the correlations were determined for the H/D experiments. Can the authors add a more detailed explanation along with an equation?

This is a good suggestion indeed. An expanded description of how we identify correlated patterns of H/D exchange has been added to the Methods. This section now includes (page 10):

"The analysis of the HDX data was a two-part process. First, compare deuterium incorporation in all conditions, the deuterium uptake was determined by monitoring shifts of the centroid peptide isotopic distribution in the mass spectra. The percent deuterium (%D) incorporated at a given condition was calculated by dividing the number of deuterium atoms incorporated (#D) by the number of deuterium atoms incorporated after 24 hours. Our investigation of the protein dynamics correlations and correlation patterns was based on the difference in %D between each region of the MoFe protein. The analysis was based on the symmetric matrix $D_{ij} = \left|d_i - d_j\right|$, which is defined in terms of the absolute values of the difference in the deuterium content %D of each peptide pairs (d_i and d_j). The correlation matrix was generated using Pearson correlation. To group patterns based on correlation of difference in the matrix elements D_{ij}, hierarchical clustering was used (1-corr formula as distance and complete linkage as agglomeration method). The number of useful clusters (distinct patterns of protein dynamics correlations) was established based on k-means clustering."

Other minor points:

Page 2: "800 Ang.^2,20" It's hard to read this because the formatting of the reference is identical to the exponent; please revise.

Now revised.

Page 17: "two show opposite trend in..." – trends

Now revised and rephrased (page 20).

Figure 9: "Swtich" in the figure should be "Switch".

Now deleted.

Reviewer #4: The manuscript proposed by Simone Raugei and co-workers deals with the investigation of the mechanical coupling in the Nitrogenase complex by means of a recent developed technique that the authors already presented in a previous publication (ref. 38 in the manuscript).
The paper is well written, and the result section is clear, maybe conclusion could be more concise. After minor revisions I recommend this manuscript for publication in PLOS computational biology.
Basically I have only some doubts from the technical part that I believe the authors will be able to dispel without problems.

If I have understood correctly, in the technique adopted by the authors one compute the covariance matrix for two crystal structures (those reported in Table S1 of the SI) reported in ref. 29. It is important here to underline for clarity some more features of these two pdb.
• Why in table S1 are reported different number of residues for 2AFK and 2AFI, if the enzyme is the same and come from the same organism (Azotobacter vinelandii)?

We thank the reviewer for raising the question. The number of residues given in Table S1 refers to the residues solved in the crystal structures. We clarified this in the caption to Table S1.

• These differences maybe are not significant for the analysis of the matrix C, but the authors should say something about…

The reviewer's comment is well taken indeed. The protein structure in the two pdb models differ by 10 residues (2AFK: 3064 residues; 2AFI: 3074 residues). Differences are limited to the protein termini, which are typically unstructured and therefore poorly resolved in the X-ray crystallography-based models and often missing in the pdb structure. Their effect in the covariance matrix and all the analysis based on it is negligible. We have provided additional text (page 6) to clarify this point:

"The number of residues in 2AFK and 2AFI PDB structures is slightly different (3064 vs. 3074). Differences are limited to the (unstructured) termini regions and are not significant for the present analysis. In order to fully utilize the crystallographic information, we included all the residues contained in the crystallographic structures. However, analyses were performed only for the common residues."

• How many residues in the two pdb?

The number of residues in the two pdb files was added in the Methods (page 6):

"Mechanical coupling analyses were conducted on the crystal structures of the ADP-bound nitrogenase complex (PDB ID: 2AFI, 3074 residues solved) and the AMPPCP-bound nitrogenase complex (PDB ID: 2AFK, 3064 residues solved)."

Regarding matrix C:

• Only Calpha are considered? Or more?

Only Cα atoms are explicitly considered (page 7):

“The normalized covariance matrix of interatomic motions **C** ≡ {C_{ij}} and the Euclidean distance matrix **D** ≡ {D_{ij}} are generated from the coordinates of the C_α atoms according to the ANM as implemented in ProDy.”

• I guess that all cofactors are considered, right?

The present discussion is based on a model whereby the cofactors are removed. However, comparison was also made with structural models with the cofactors included as described in an earlier publication [PNAS **113** (2016) E5783-E5791]. Differences are negligible. A comment was added to the Methods (page 6):

“In the present study, the explicit presence of the cofactors was neglected. Inclusion of the cofactors as done in earlier studies yields negligible differences in the covariance matrix, and the analysis based on it does not change.”

Then, why the authors decided to consider this approach instead of a molecular dynamic (MD) approach? We all know that MD for such metal-protein systems is quite demanding, but a word on this issue should be beneficial for the readers.

The reviewer raised a very good point. Covariance analysis from atomistic molecular dynamics simulations of the nitrogenase complex in an aqueous environment was indeed our first choice. In fact, we have performed a multi-microsecond MD simulation of both the ATP- and ADP-bound complexes. Unfortunately, because of the extremely large size of the system, the low frequency, large amplitude motions of the complex are still far from being converged. A comment has been added to the Methods (page 6):

“Coarse-grained ANM calculations are definitely more amenable for the type of analysis presented here than a principal component analysis based on molecular dynamics trajectory. Indeed, the size of the nitrogenase complex poses serious limitations on the convergence of long-time, large-amplitude modes, which are crucial for the long-range communication.”

Finally, the authors validated their results based on the H / D exchange experiments, a fact that gives value to the manuscript. However C matrix is computed on just two structures, and I would like that the authors could comment this in light of the standard MD PCA analysis made on a complete trajectory.

Again, unfortunately, the very large size of the system (the simulation cell contains ~800,000 atoms, ~48,000 of which are the protein complex) does not allow for a reliable PCA analysis based on MD trajectories. That said, under the reasonable assumption that the complete H/D exchange discussed in the text correspond to the D content after full ATP hydrolysis, we believe it is

meaningful to compare correlation in the H/D exchange for different parts of nitrogenase with the mechanical coupling inferred from the ATP- and ADP-bound structures.
