# Supplementary material for: Mechanical coupling in the nitrogenase complex
Source: PLoS Comput Biol. 2021 Mar 4;17(3):e1008719. doi: 10.1371/journal.pcbi.1008719 (PMC7963043; doi:10.1371/journal.pcbi.1008719)
Supplement: Attachment — Submitted filename: Response_20201219.pdf [file pcbi.1008719.s012.pdf]

Reviewer's Responses to Questions

Comments to the Authors:

Please note here if the review is uploaded as an attachment.

Reviewer #1: The current revision is not satisfactory for reasons below and should be amended.

Authors:
Here we would like to reiterate that the method we have developed was designed to filter out pairs of residues whose covariant motion is purely coincidental and evaluate potential communication pathways between distant region of a protein complex. The major difference between our M-matrix analysis and a simple covariance analysis of motion, is that if two residues have correlated motions but do not have an efficient path for mechanical coupling between them (large geodesic distance in the covariance space), a small (poorly coupled) mechanical coupling (as described by the M matrix) will be produced. In this way, geodesic weighting filters out motions that are merely coincidental. Clearly, the existence of a pathway is a necessary but not sufficient condition for causality. This is actually an important point, which was added in the revised manuscript (page 23).

Reviewer:
Explain directly in the work what "mechanical coupling between them (large geodesic distance in the covariance space)" means in terms of protein dynamics that underlies allosteric communication.

We thank the reviewer for pointing out parts of our article that were still unclear. We explained the meaning of mechanical matrix in terms of the allosteric pathways on page 7. To further clarify the concepts behind the mechanical coupling matrix and the corresponding weighting trough the geodesic distance, we added a few additional comments on page 7:
"In the proposed mechanical coupling analysis, we filter out the purely coincidental correlated or anticorrelated motions inferred from the covariance analysis by weighting each element C_{ij} of the covariance matrix by the geodesic distance matrix in the covariance space between residue *i* and residue *j*. By doing so, we focus on how the motions of the nitrogenase complex about one equilibrium conformation allow for the communication between the distant sites. In this spirit, "mechanical coupling" here indicates that two residues or two regions not only have correlated dynamics, but, more importantly, there is an efficient allosteric communication pathway connecting them (*i.e.*, short geodesic distance). However, it does not necessarily indicate any functional role, whose assessment is clearly beyond the coarse-grained description of the protein adopted here. "

Authors:
"We like to emphasize that correlation or communication here does not imply causality. In fact, strong mechanical coupling indicates an efficient coupling pathway connecting two residues or regions which show correlated or anti-correlated motions. Clearly, the existence of a pathway is

a necessary but not sufficient condition for causality. A study of effects of suppression or delay of the motion of one region on the motion of the other will be necessary to consider causality."

Reviewer:
It should be directly stated in teh above paragraph that causality can only be quantified via analysing the enegetics of allosteric signalling with corresponding references (PMID: 26939022, PMID: 29912863, PMID: 30439587).
We thank the reviewer for the comment. We clarify this point and added the suggested references in the text (page 23):
"The study of the effect of suppression or delay of the motion of one region on the motion of the other, and/or the analysis of the energetics of allosteric signaling have been recently proposed to address causality of the correlation."

Authors:
We also realize that the language adopted in the manuscript was not clear. In particular, as originally discussed, the concept of energy transfer could have led to misinterpretations. We used the term energy transfer to indicate instantaneous flow of kinetic (thermal) energy mediated by protein vibrations (normal modes). We have revised and consolidated the language to address this point. For example, where appropriate, "energy" is changed to "information" and "energy flow" changed to "communication".

Reviewer:
"information", "structural or dynamic information", "information transfer" and other similar usage of the word "information" is not correct and should be changed throughput the text.

We thank the reviewer for this remark. We originally adopted the term "information" because that it the term adopted in the graph-theory we employed. We realize that we should use terms that are commonly adopted to describe allostery. Now "information" on page 8 and 17 is changed to "signaling" or "allosteric signaling".

Authors:
There was an early study of Liao and Beratan that reported coarse grained normal mode analysis of the nitrogenase proteins as well (see Biophysical Journal 87 1369–1377 (2004)). It would be interesting to compare the results of the two studies and to place the early albeit less comprehensive study into the context of this modern analysis, if possible.
We thank the Reviewer for pointing out the work by Liao and Beratan. We compare their results with ours in the Results and Discussion of the revised manuscript (page 12).

"Within each Fe protein, the P-loops are coupled to the protein-protein interface and the [4Fe-4S] cluster through the switch region I and II, respectively. Liao and Beratan discussed this coupling in their computational analysis of the isolated Fe protein. Using a coarse-grained model, they identified correlated regions in the Fe protein and reported that residues in the P-loops and switch regions are relatively rigid in the slowest normal mode, which can be important for function-relevant conformational changes. As we will discuss below, we show that there is indeed

an efficient communication pathway between these regions, which, in the nitrogenase complex, extends up to the interior of the MoFe protein."

Reviewer:
Since authors compared their results with those od Liao and Beratan at the request of second reviewer, I do not understand why they did not obtain the picture of the energetics of allosteric signalling using AlloSigMA cited in my previous review. In this case authors would be able to talk about causality and energetics avoiding to use wrong terminology as noted above.

We appreciate the suggestion from the Reviewer. AlloSigMA is certainly a powerful tool to address K-type allostery (substrate affinity is changed upon binding of an effector or mutation of residues). However, that is not the focus of the present work. In this work, we investigated (1) what parts of the nitrogenase complex are coupled together, through equilibrium vibrational motions and not in response to the binding of an effector or mutation of residues, and (2) what are the underlying coupling pathways. In addition, it is not clear what regions in the nitrogenase are the functional and regulatory sites for the initial electron transfer event so a full scan on the complex is necessary, while the size of the nitrogenase (over 3,000 residues) exceeds the limits of the AlloSigMA webserver for a full allosteric signaling map. As for the terminology that we originally used in our manuscript, we are confident that we have addressed it properly as indicated above.

Reviewer #2: The authors have addressed all referee concerns and the paper is suitable for publication.

Reviewer #3: The authors have sufficiently addressed my concerns.
